# The disordered structure of sparsentan: energy calculations for com­peting chain con­for­mations

**DOI:** 10.1107/S2053229625007181

**Published:** 2025-08-26

**Authors:** Thomas Gelbrich, Kristaps Saršüns, Doris E. Braun

**Affiliations:** a Christian Doppler Laboratory for Advanced Crystal Engineering Strategies in Drug Development, Innrain 52c, Innsbruck, 6020, Austria; b Institute of Pharmacy, University of Innsbruck, Innrain 52c, Innsbruck, 6020, Austria; Oak Ridge National Laboratory, USA

**Keywords:** crystal structure, disorder, energy calculations, con­for­mation, pharmaceutical, flexible molecule, sparsentan, SST

## Abstract

The crystalline form of sparsentan (SST) (systematic name: 2-{4-[(2-butyl-4-oxo-1,3-di­aza­spiro­[4.4]non-1-en-3-yl)meth­yl]-2-(eth­oxy­meth­yl)phen­yl}-*N*-(4,5-dimethyl-1,2-oxazol-3-yl)benzene­sulfonamide), C_32_H_40_N_4_O_5_S, was produced and characterized using single-crystal and powder X-ray diffraction, differential scanning calorimetry (DSC), thermogravimetric analysis (TGA) and IR spectroscopy. The SST mol­ecule displays positional disorder in three different sections. Viability tests of alternative disorder models involved the calculation of energetic contributions to analyse each possible mol­ecular con­for­mation within its crystal environment and identify the energetically most favourable con­for­mations in the lattice.

## Abstract 2

A mol­ecule of sparsentan displaying a com­plex disorder of multiple sections was modelled and inter­preted with the assistance of energy calculations.

## Introduction

Sparsentan (SST, Scheme 1) is a dual endothelin type A (ETA) receptor and angiotensin II type 1 (AT1) receptor antagonist (DEARA), developed by Travere Therapeutics and marketed under the brand name Filspari. It is used for the treatment of IgA nephropathy and focal segmental glomerulosclerosis (FSGS) (Syed, 2023 ▸; Zhang *et al.*, 2020 ▸). By blocking the ETA receptor, this drug reduces vasoconstriction, inflammation and fibrosis, while its AT1 receptor antagonism lowers blood pressure and protects kidney function. This dual mechanism is effective for treating kidney diseases, such as focal segmental glomerulosclerosis (FSGS) and IgA nephropathy (IgAN), as proteinuria is reduced and disease progression slowed (Kohan *et al.*, 2024 ▸). The SST mol­ecule is highly flexible and contains seven hy­dro­gen-bond acceptors, one hy­dro­gen-bond donor and 12 torsion angles (Murugesan *et al.*, 2002 ▸). Its mol­ecular flexibility predisposes sparsentan to the formation of an amorphous phase (Macikenas *et al.*, 2019 ▸). An amorphous phase typically dissolves faster than its stable crystalline counterpart, but is generally less stable over time, which can potentially lead to performance variability (Desiraju, 2007 ▸). Although limited structural data for this phase have been disclosed (Murugesan *et al.*, 2005 ▸), the detailed structural and con­for­mational information which are necessary to understand its properties and behaviour have not been available so far. Therefore, we have carried out a com­prehensive crystallographic study to establish the solid-state characteristics of the SST mol­ecule.

Flexible mol­ecules can adopt a range of energetically viable con­for­mations whose specific crystal packing preferences may then result in the formation of polymorphs, *i.e.* a single mol­ecule crystallizes in multiple crystal forms with distinct mol­ecular packing arrangements and physical properties (Tang *et al.*, 2021 ▸). The study of flexible mol­ecules is therefore an important topic in materials science, pharmaceutical research and crystal engineering. In addition, mol­ecular flexibility may also be linked with the observation of structural disorder in certain crystals. Many mol­ecular crystals and more than 20% of the crystal structures deposited in the Cambridge Structural Database (CSD) exhibit some form of disorder (Groom *et al.*, 2016 ▸; Linden, 2023 ▸). The accurate refinement of these structures depends critically on the available diffraction data, as only high-quality data enable a reliable determination of the mol­ecular geometry and a sufficient resolution of the disordered regions, and weak data will lead to inferior results (Diederichs, 2016 ▸). In this context, energy calculations that are aimed at identifying the energetically most favourable con­for­mations within the lattice have become a valuable tool for a more reliable disorder refinement. For instance, energy minimization techniques like the ‘mol­ecule-in-cluster’ approach allow the analysis of each of the possible mol­ecular con­for­mations within its crystal environment, refining the model based on the most stable con­for­mations (Dittrich, 2021 ▸). By com­putationally optimizing each mol­ecular arrangement and applying targeted restraints, the alignment between the model and the experimental data is improved, and thus the accuracy and precision of disorder refinement in structural analysis is enhanced (Müller, 2021 ▸). In the present study, the crystal structure refinement revealed multiple disordered fragments within the SST mol­ecule. Advanced techniques, including energy calculations (Clark *et al.*, 2005 ▸), were applied to assess the viability of alternative disorder geometries and also to evaluate which of these are likely to co-exist in individual mol­ecules.
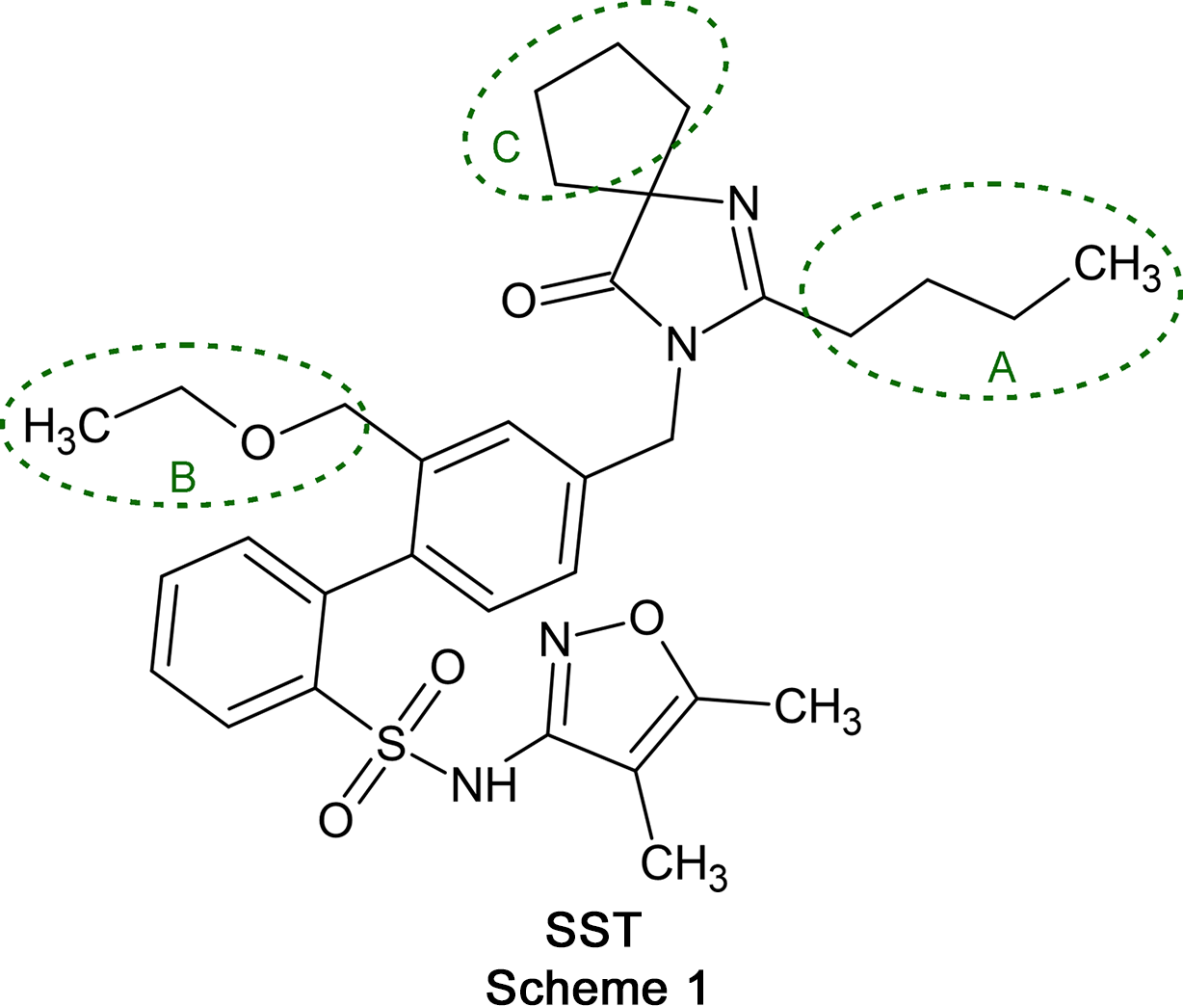


## Experimental

### Materials

Amorphous SST was obtained from Taros Chemicals. This substance was recrystallized prior to use in further experiments (details of the recrystallization method are provided below). Analytical grade solvents were procured from com­mercial suppliers.

### Preparation of crystalline SST

Amorphous SST (1 g) was dissolved in 5 ml of iso­propanol and 5 ml of water was added dropwise to the mixture. The resulting mixture was then warmed to 40 °C to produce a clear solution. This solution was allowed to cool and kept at room tem­per­a­ture, resulting in the formation of white prismatic crystals suitable for single-crystal structure determination. These crystals were filtered off, washed with a small amount of a 2:1 iso­propanol–water mixture and dried to yield a white crystalline solid.

### Single-crystal structure refinement

Crystal data, data collection and structure refinement details are summarized in Table 1 ▸. All H atoms, except for those in disordered fragments, were identified in difference maps. Methyl H atoms were idealized and included as rigid groups allowed to rotate but not tip (C—H = 0.98 Å), with *U*_iso_(H) parameters set to 1.5*U*_eq_(C) of the parent atom. H atoms bonded to secondary CH_2_ and tertiary CH atoms (C—H = 0.99 Å), and H atoms in aromatic groups (C—H = 0.95 Å) were positioned geometrically, with *U*_iso_(H) values set to 1.2*U*_eq_(C) of the parent atom. The H atom of the NH group was refined with a restrained bond length [N6—H6 = 0.88 (1) Å] and its *U*_iso_(H) parameter was refined freely.

Several sections of the SST mol­ecule were found to exhibit positional disorder, *i.e.* the butyl group (–C39—C40—C41—C42; labelled **A** in Scheme 1), the eth­oxy­methyl group (–C24—O25—C26—C27; labelled **B**) and the cyclo­pentyl ring (C34–C37; labelled **C**). A combination of restraints on chemically equivalent 1,2- and 1,3-distances, and restraints on anisotropic displacement parameters was applied in the refinement of the non-H-atom positions in the disordered parts of the mol­ecules. The refined final occupancies were **A1**/**A2** = 0.804 (5):0.196 (5), **B1**/**B2**/**B3** = 0.597 (3):0.223 (3):0.180 (2) and **C1**/**C2** = 0.571 (18):0.429 (18). A detailed description of the disorder model is given in Section 3.2 below.

### Periodic electronic structure calculations

Six ordered structure models based on the disorder fragments in sections **A** and **B** of the SST mol­ecule (Scheme 1) were generated. These models represented the initial configurations for periodic electronic structure optimization using *CASTEP* (Version 23.1; Clark *et al.*, 2005 ▸) to generate optimized atomic positions and lattice parameters. The Perdew–Burke–Ernzerhof (PBE) generalized gradient approximation (GGA) exchange-correlation density functional (Perdew *et al.*, 1996 ▸) was applied, along with ultrasoft pseudopotentials (Vanderbilt, 1990 ▸) and the MBD* semi-empirical dispersion correction (Tkatchenko *et al.*, 2012 ▸). The *k*-point grid was chosen to maintain a maximum spacing of 2π × 0.07 Å^−1^, with a basis set cut-off energy of 780 eV. Convergence criteria were set to an energy precision better than 2 × 10^−5^ eV per atom, atomic displacements within 1 × 10^−3^, maximum forces below 1 × 10^−3^ Å and maximum stresses below 0.1 GPa. The main com­ponent of the cyclo­pentyl ring (section **C** in Scheme 1) was used for all models, as the calculations were carried out to investigate the disordered chain con­for­mations.

### Pairwise inter­molecular energy calculations

*CrystalExplorer* (Version 17; Mackenzie *et al.*, 2017 ▸; Spack­man *et al.*, 2021 ▸) and *GAUSSIAN16* (Frisch *et al.*, 2019 ▸) were used to calculate the pairwise inter­molecular energies for the PBE-MBD*-generated structure models of SST within a 3.80 Å radius, using the B3LYP/6-31G(d,p) wavefunction.

## Results and discussion

### Mol­ecular geometry and hy­dro­gen bonding

**Figure 1:**
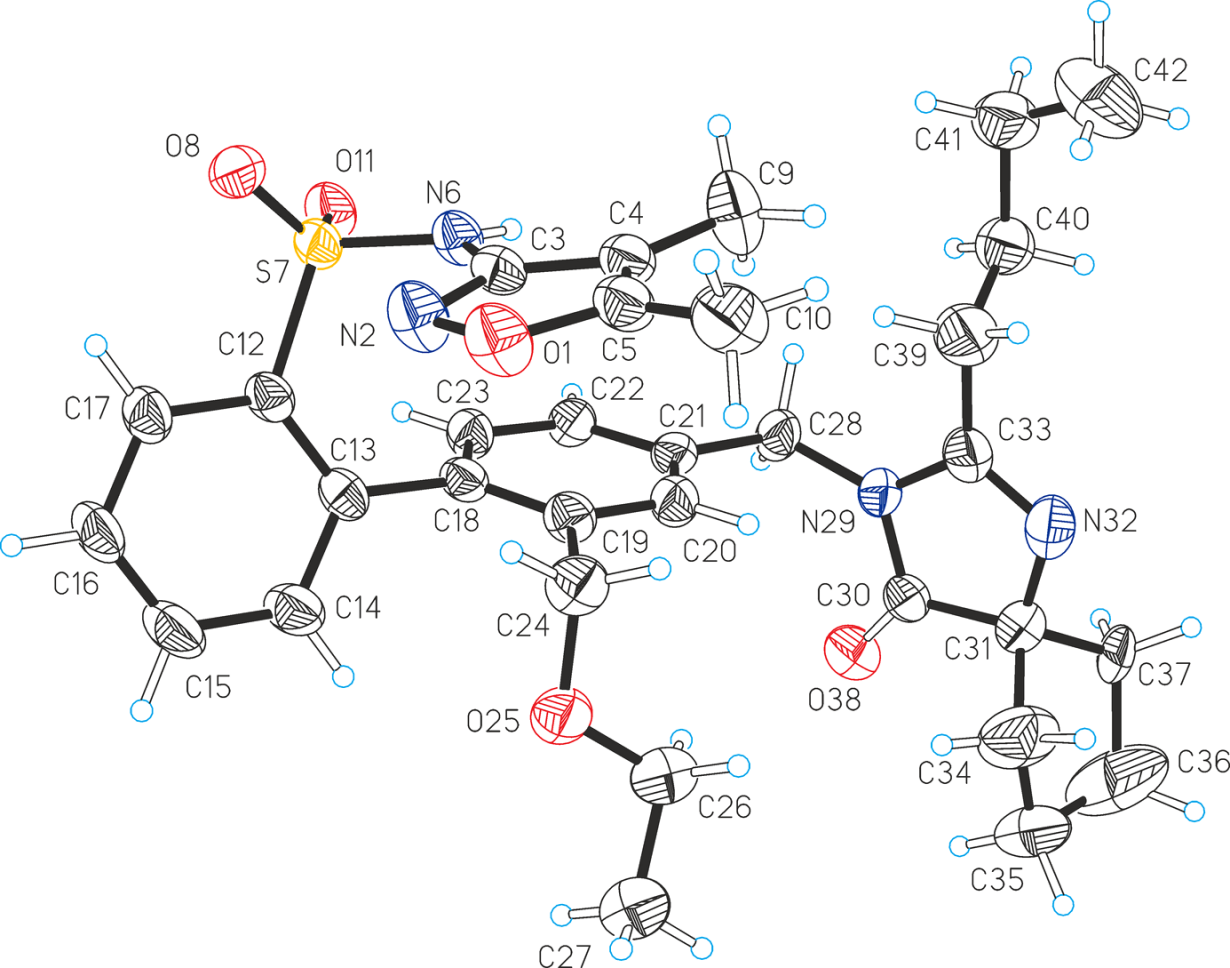
The mol­ecular structure of SST, with displacement ellipsoids drawn at the 50% probability level and H atoms drawn as spheres of arbitrary size (minor disorder has been omitted for clarity).

**Figure 2:**
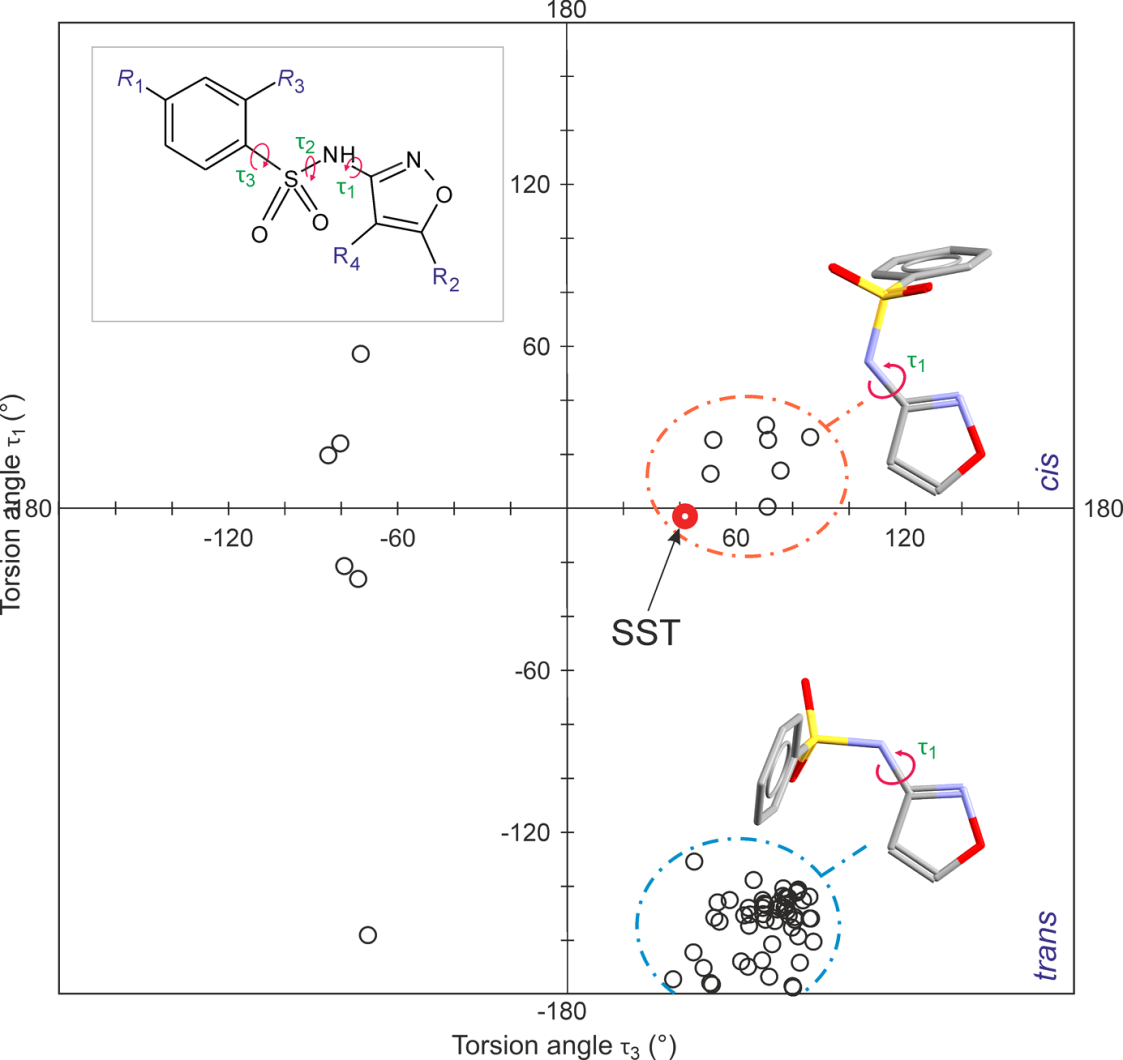
Survey of the experimental con­for­mations of the *N*-(1,2-oxazol-3-yl)ben­zene­sulfonamide fragment characterized by torsion angles τ_1_–τ_3_ (inset). The τ_3_*versus* τ_1_ plot shows two clusters which correspond to preferred con­for­mations.

The asymmetric unit contains a single SST mol­ecule (Fig. 1 ▸). The C12—C13—C18—C23 torsion angle, corresponding to the twist between the two central arene rings, is −69.7 (3)°. The C28—N29 bond is slightly out of the mean plane formed by atom C28 and the adjacent arene ring [C20—C21—C28—N29 = 14.6 (3)°], and the angle between the latter mean plane and that defined by atom C28 and the neighbouring imidazole ring (N29/C30/C31/N32/C33) is 82.33 (8)°. In the *N*-(1,2-oxazol-3-yl)benzene­sulfonamide fragment, the N2—C3 bond of the oxazole ring and the S7—C12 bond adopt a *cis* arrangement, and the central C3—N6—S7—C12 chain displays a *gauche* con­for­mation, corresponding to values of 3.2 (3), −60.5 (2) and −41.7 (2)° for the three essential torsion angles N2—C3—N6—S7 (τ_1_), C3—N6—S7—C12 (τ_2_) and N6—S7—C12—C13 (τ_3_). The most recent version of the Cambridge Structural Database (CSD, Version 6.00; Groom *et al.*, 2016 ▸) contains another 67 examples of the same mol­ecular fragment in 55 different crystal structures, and the values of the torsion angles τ_1_–τ_3_ (Fig. 2 ▸, inset) were collected for each of these examples (Table S1 of the supporting information). As two inversion-equivalent sets of torsions are present in each of these structures, the values of τ_1_, τ_2_ and τ_3_ were normalized so that τ_2_ ≥ 0° in order to facilitate a meaningful com­parison. The obtained geometrical parameters are listed in Table S1 of the sup­porting information. All values for the central angle τ_2_ lie within a narrow range between 50.1 and 86.1°, *i.e.* the central C—N—S—C chain is always *gauche* and its con­for­mational flexibility is small. Moreover, a plot of τ_1_ values against τ_3_ angles reveals two distinct densely populated clusters of data points in the narrow range 41.7 ≤ τ_3_ ≤ 86.5°. The corresponding τ_1_ values approach 180° in the first cluster (encircled in blue in Fig. 2 ▸), indicating a *trans* orientation of the S—C bond relative to the N—C bond of the oxazole ring. By contrast, τ_1_ values are close to 0° in the second cluster (encircled in red in Fig. 2 ▸), which corresponds to a *cis* con­for­mation and also contains the data point for SST. Therefore, the τ_1_ torsion describes a section of the mol­ecule which is typically planar and gives rise to two distinct geometries which are related by a 180° rotation about the central N—C bond.

**Figure 3:**
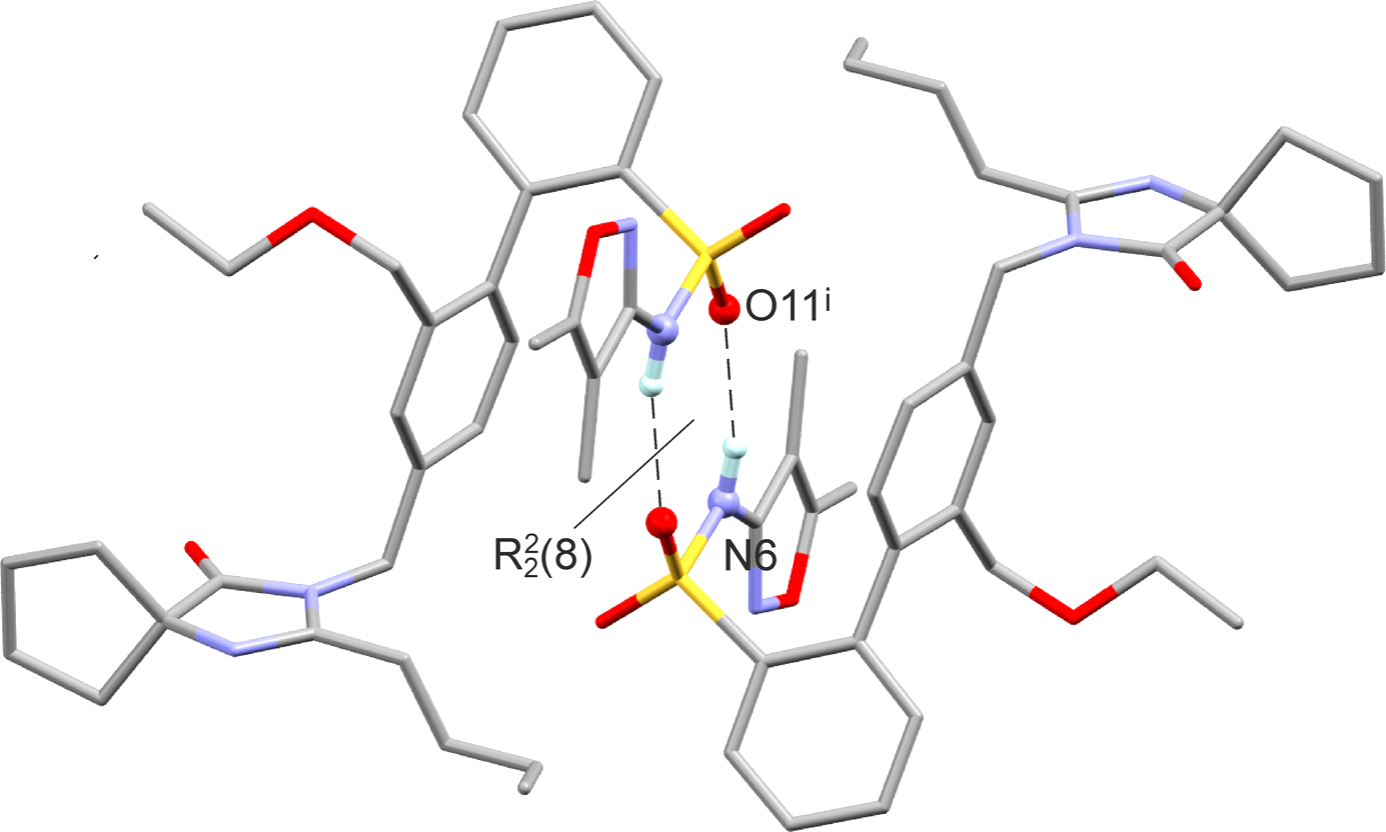
N—H⋯O hy­dro­gen-bonded dimer (minor disorder and H atoms bonded to C atoms have been omitted for clarity). [Symmetry code: (i) −*x* + 1, −*y* + 1, −*z* + 1.]

The SST mol­ecule contains seven potential acceptor sites for hy­dro­gen bonds and one hy­dro­gen-bond donor group, *i.e.* the NH group of the sulfonamide fragment. The latter forms an N6—H6⋯O11^i^ inter­action with a sulfonyl O atom of a neighbouring mol­ecule (see Table 2 ▸ for symmetry codes). The resulting centrosymmetric dimer (Fig. 3 ▸) displays an 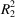
(6) ring (Etter *et al.*, 1990 ▸; Bernstein *et al.*, 1995 ▸). Within this dimer, there is also a short inter­molecular contact, C28—H28*B*⋯O8^i^, with an H⋯O separation of 2.46 Å, which involves the second sulfonyl O atom and the CH_2_ group linking the substituted imidazole ring with the arene ring (Table 2 ▸). The same CH_2_ group is additionally engaged in a significant inter­action with the carbonyl group of a third mol­ecule, C28—H28*A*⋯O38^ii^, where the H⋯O distance is 2.40 Å.

### Disorder model

In the case of the disordered **B** chain, the initial refinement was also carried out with a two-com­ponent model. The re­sulting disordered chain geometries displayed sensible 1,2- and 1,3-distances. The corresponding occupancy ratio was 0.7:0.3, *R*[*F*^2^ > 2σ(*F*^2^)] = 0.066 and *wR*(*F*^2^) = 0.205. However, this model displayed some unusual features, especially with regard to the position of the C27′ methyl group of the minor com­ponent (see Fig. S6 of the supporting information). In addition to an unexpected large spatial separation between alternative methyl-group positions (C27⋯C27′ = 1.94 Å), the C27′ methyl group was in close proximity to the C18–C23 ring of a second mol­ecule with C27′⋯C23^iii^ = 2.63 Å [symmetry code: (iii) −*x* + 1, −*y* + 2, −*z* + 1], resulting in several atypically short inter­molecular distances. This situation could not be improved by the application of anti-bumping restraints. Moreover, the region of the disordered **B** chain also contained significant residual electron-density peaks, with Δρ_max_ = 1.02 e Å^−3^ at a distance of 2.64 Å from C23^iii^.

Additionally, energy calculations carried out on the four theoretical ordered crystal structures containing optimized mol­ecular geometries representing possible **A**/**B** combinations indicated a significant disadvantage associated with the minor **B** com­ponent (see Fig. S7 of the supporting information).

**Figure 4:**
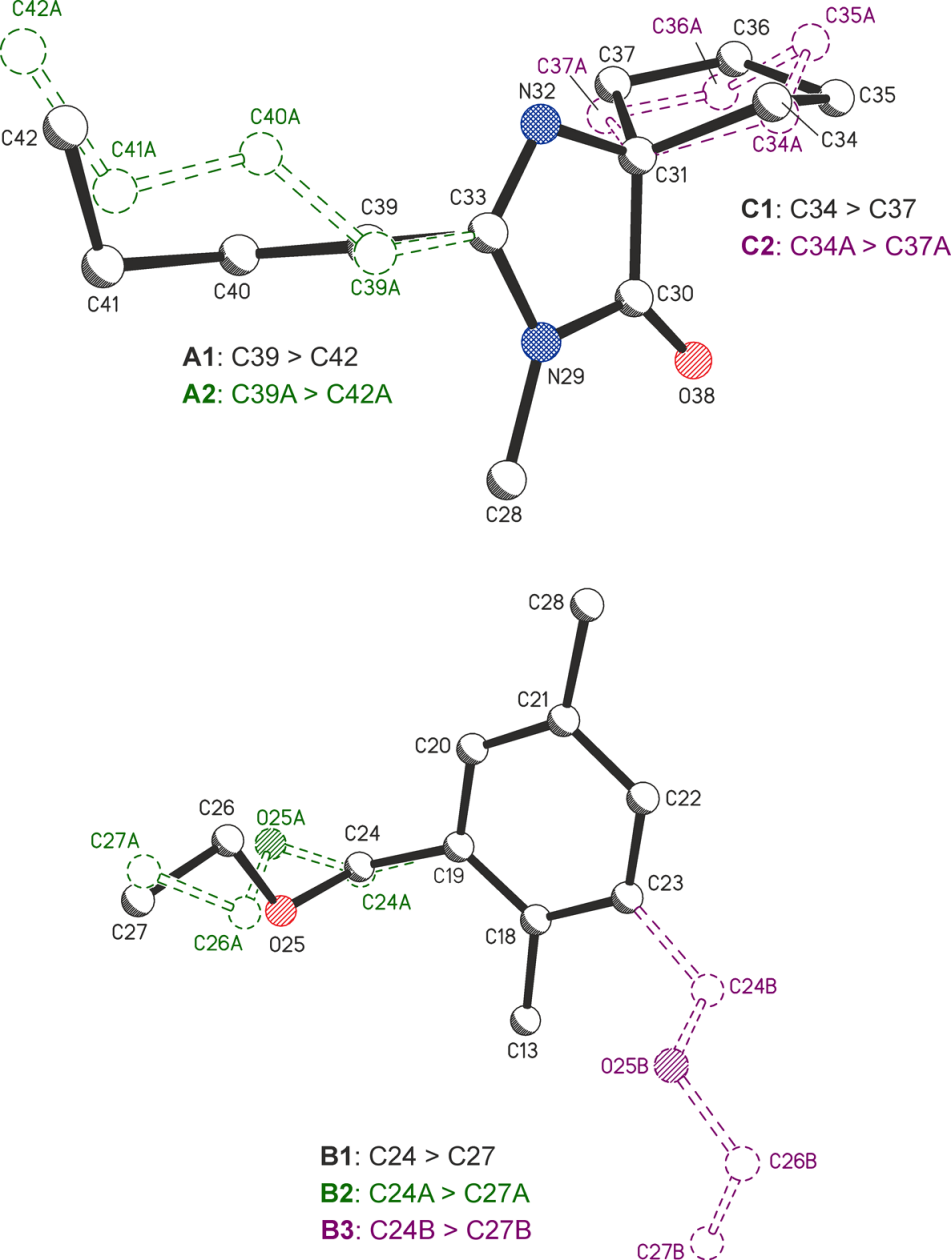
Detailed views of the alternative chain con­for­mations of the disorder com­ponents **A1**/**A2** and **C1**/**C2** (top), and **B1**/**B2**/**B3** (bottom; H atoms have been omitted for clarity).

The search for a better model then let us consider the possibility of a third disorder com­ponent **B3** of the chain (–C24—O25—C26—C27), which results from the rotation of the arene ring about the C13—C18⋯C21—C28 axis by ap­proximately 180° (see Fig. 4 ▸). The geometry of the first dis­order com­ponent **B1** was largely unchanged in this new three-way-split model. The main difference between **B1** and the second com­ponent **B2** is a 44° rotation about the C19—C24 bond (see Table S8 of the supporting information). As expected, the alternative positions of the two methyl groups lie in very close proximity, *i.e.* C27⋯C27*A* = 0.371 (16) Å. The final occupancies of **B1**, **B2** and **B3** were 0.597 (3), 0.223 (3) and 0.180 (2), respectively. The presence of a **B3** chain in a given mol­ecule means that a particular neighbouring mol­ecule to which it is related by the inversion operation (−*x* + 1, −*y* + 2, −*z* + 1) must also contain the **B3** con­for­mation, *i.e.* the **B3** geometry of one mol­ecule is geometrically incom­patible with a **B1** or **B2** chain in the other. The refined site occupancy of 0.804 for the major **A1** com­ponent is similar to the sum of the occupancies of 0.820 for **B1** and **B2**, but it cannot be ascertained from diffraction data how the occupancies in sections **A** and **B** of the SST mol­ecule are correlated with one another. This topic was investigated further with energy calculations, discussed in the following section, which were also used to establish the viability of the obtained chain con­for­mations.

The con­for­mations **C1** and **C2** of the disordered cyclo­pentyl ring were analysed using *PLATON* (Spek, 2020 ▸). Ring-puckering parameters (Cremer & Pople, 1975 ▸) of *q* = 0.421 (13) Å and ϕ = 28 (2)° obtained for the major com­ponent indicate an inter­mediate geometry between C34-envelope and C31/C34-twist. The minor com­ponent (Fig. 4 ▸) displays a con­for­mation between C36*A*-envelope and C36*A*/C35*A*-twist, resulting in ring-puckering parameters of *q* = 0.338 (19) Å and ϕ = 279 (3)°.

### Energy calculations

**Figure 5:**
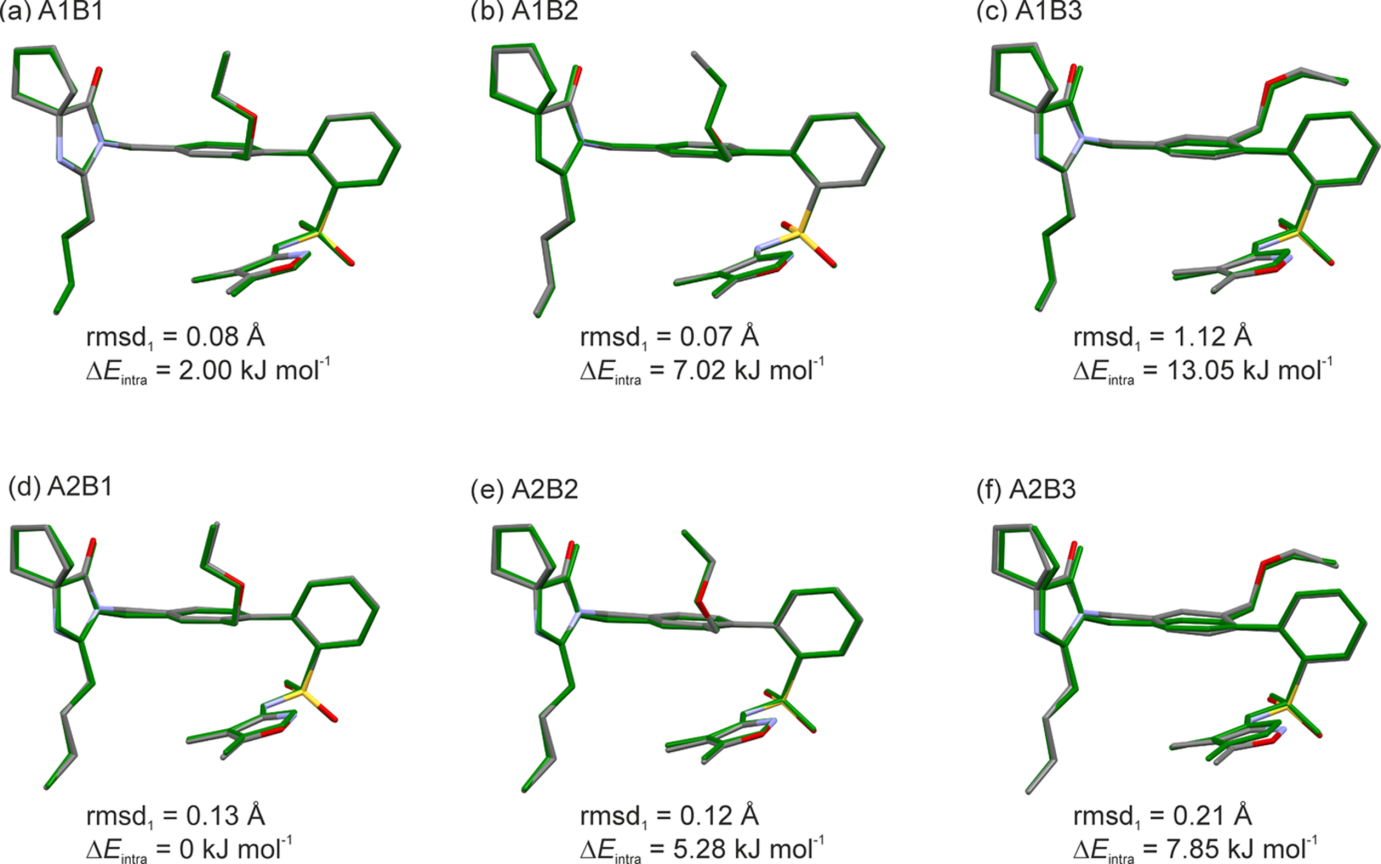
Overlay of the experimental con­for­mations (coloured by element) with the con­for­mations observed in the optimized structures (in green). Rmsd_1_ and intra­molecular energy differences (Δ*E*_intra_) were calculated at the B3LYP/6-31G(d,p) level of theory. Δ*E*_intra_ values are reported relative to the lowest energy among the six con­for­mations.

To further investigate the disorder in the –CH_2_CH_2_CH_2_CH_3_ (section **A** in Scheme 1) and –CH_2_OCH_2_CH_3_ (section **B**) groups, six independent ordered structural models were generated, each starting from one of the disorder sites identified in the experimental structure. These structures were then optimized (PBE-MBD*), with the experimental lattice parameters and atomic positions allowed to minimize. The minimizations revealed that the six initial models did not con­verge to a single structure. Instead, each of the possible positions for both disordered groups yielded a unique minimum on the lattice energy landscape, indicating that the disorder in the **A** group is not linked to the disorder in the **B** group, and *vice versa*. Each of the positions of the **A** and **B** groups was well reproduced in the PBE-MBD* structures, as shown by the overlay of experimental and PBE-MBD* con­for­mations (Fig. 5 ▸).

The intra­molecular energy differences (Δ*E*_intra_) for the six con­for­mations were estimated at the B3LYP/6-31G(d,p) level of theory. **A2B1** was identified as the lowest-energy con­for­mation among the six (calculated in the gas phase), although it was only 2.00 kJ mol^−1^ lower than **A1B1**. Therefore, the energy difference between the two **A** chain orientations is relatively small com­pared to the energy differences between the **B** orientations, which were estimated to be in the range 5–11 kJ mol^−1^.

Less favourable intra­molecular energies can be offset by stronger inter­molecular inter­actions within the crystal structure. A com­parison of the lattice energies (PBE-MBD*) of the six distinct orientations (Table 3 ▸) in the SST structure revealed that the three structures adopting the **A1** orientation are lower in energy than the three **A2**-based structures. In the experimental structure, the disorder ratio was refined to 0.45:0.55, with **A2** being only slightly favoured. Adding the **B** orientations to the com­parison revealed that **B1** is favoured over **B2** and **B3**, which is clearly reflected in the disorder ratio of 0.60 for **B1**, com­pared to 0.22 and 0.18 for the remaining two orientations. Overall, the structure optimizations revealed that **A1B1** might be the most stable of the six models based on lattice energy calculations. However, with the exception of the **A2B2** model, all are within 10 kJ mol^−1^. The calculations de­mon­strate that numerous orientations are feasible for forming low-energy structures, and adding entropic contributions is expected to further stabilize the structures. This helps rationalize the high tendency toward disorder in the SST structure. All orientations were well reproduced in the models, with the **B3** orientation, a minor orientation involving a 180° flip of the bi­phenyl Ph–Ph dihedral, showing a slightly higher rmds_15_ value than the other structures (Table 3 ▸).

**Figure 6:**
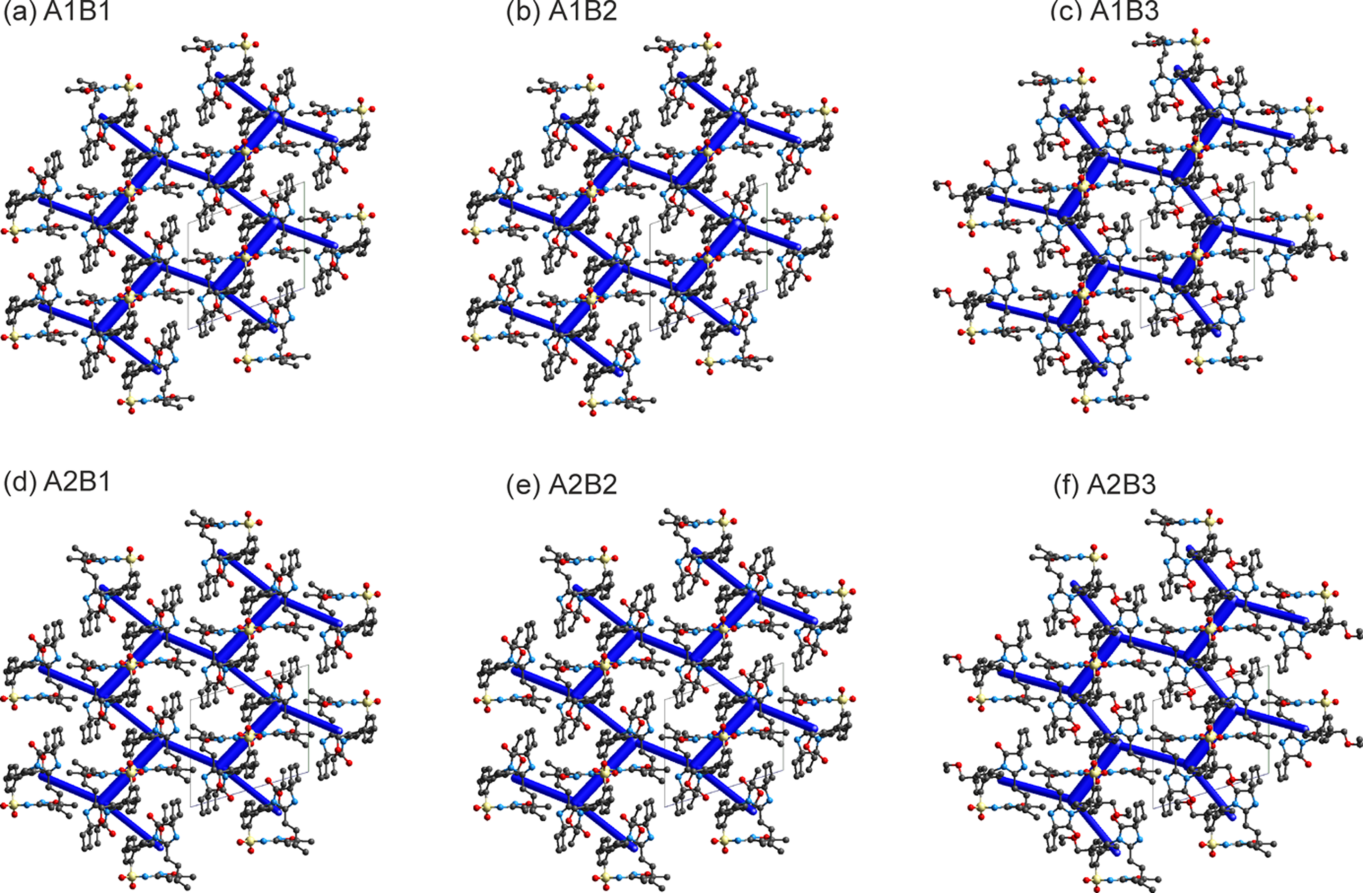
Energy framework diagrams (total energy), illustrating that all six tested sparsentan models result in the same order of the strong pairwise inter­actions. The energy scale factor is 50. Stabilizing contacts are shown in blue and the thickness corresponds to the strength. Pairwise inter­action energies less than 20 kJ mol^−1^ and H atoms have been omitted for clarity. The packing diagrams are displayed along the respective crystallographic *a* axes.

In addition to calculating the lattice energy differences between the models, we also com­puted the pairwise inter­molecular inter­action energies for the PBE-MBD* structures. The six optimized structure models exhibit identical packing arrangements (Fig. 6 ▸), differing only in the torsional variations of the two flexible groups. The 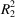
(8) dimer motif was identified as the strongest pairwise inter­molecular inter­action, with an inter­action energy between −127.8 and −150.1 kJ mol^−1^. This inter­action is stabilized not only by significant electrostatic contributions but also by dispersion forces. Sparsentan, which has a single hy­dro­gen-bond donor group, but multiple aromatic rings and flexible alkyl chains, allows for the formation of relatively strong aromatic inter­actions and close contacts. The second and third strongest inter­actions fall within the −92.5 to −72.9 kJ mol^−1^ range (Tables S3–S6 of the supporting information) and, despite their strength, they do not involve classical strong hy­dro­gen bonds, highlighting the crucial role of dispersion forces in stabilizing the crystal lattice. The fact that each of the six models remained at a local minimum without transitioning to another arrangement sug­gests a potential for disorder, often seen in com­puted crystal energy landscapes where major and minor com­ponents appear as separate structures (Hunnisett *et al.*, 2024 ▸). The applied lattice energy minimization models do not account for entropic contributions, which are expected to stabilize the structure and, therefore, support the experimentally observed disorder.

### Characterization of the crystalline phase of SST

#### Powder X-ray diffraction (PXRD)

**Figure 7:**
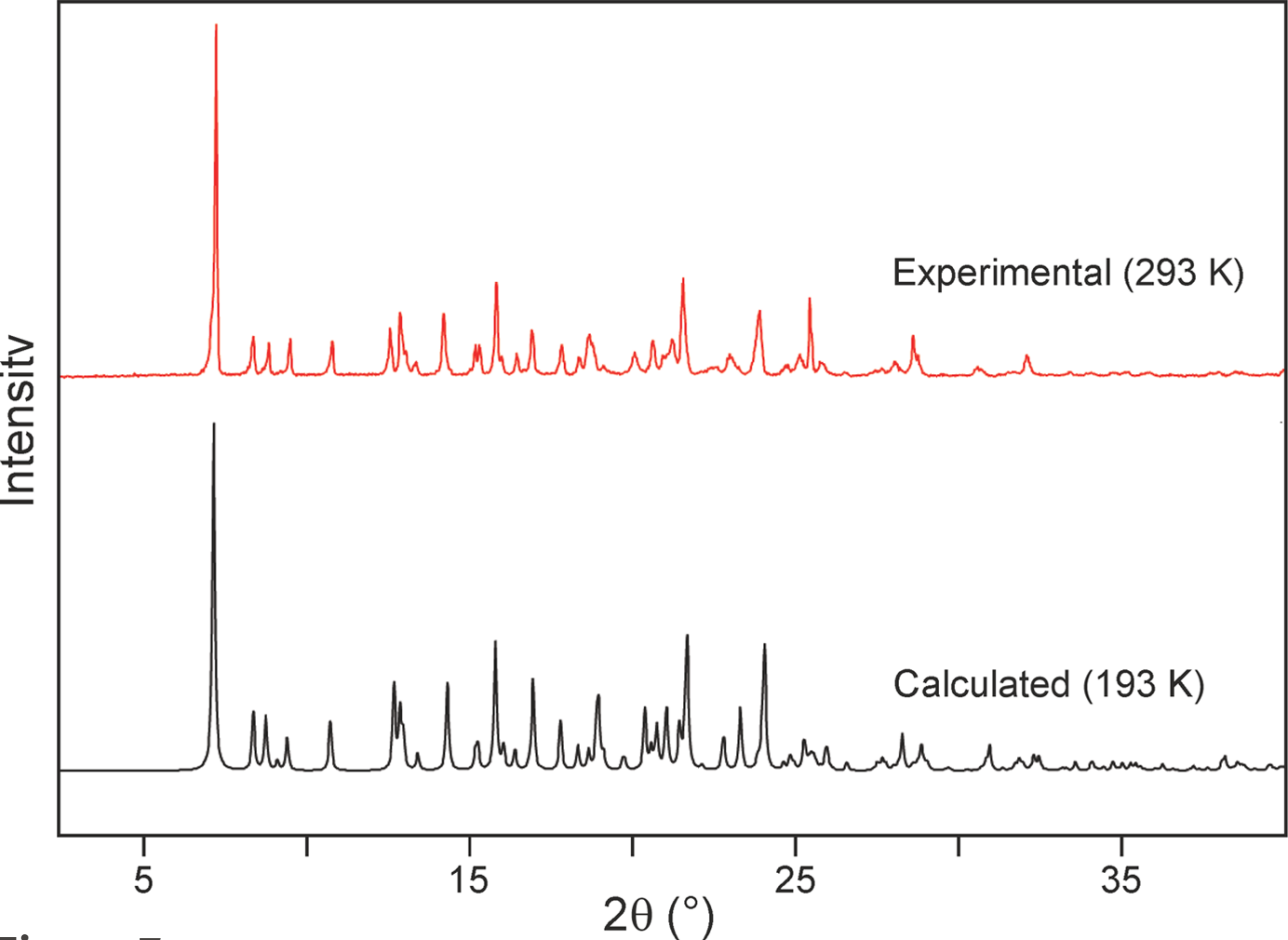
Experimental PXRD pattern of SST (top) and a simulated pattern derived from the single-crystal structure (bottom).

The experimental room-tem­per­a­ture PXRD pattern of a sample of SST obtained *via* cooling crystallization from aceto­nitrile matches a corresponding pattern (193 K) calculated with *Mercury* (Macrae *et al.*, 2020 ▸) from the crystal structure data (Fig. 7 ▸). Slight differences in peak positions are due to different tem­per­a­ture conditions.

#### Thermal analysis

**Figure 8:**
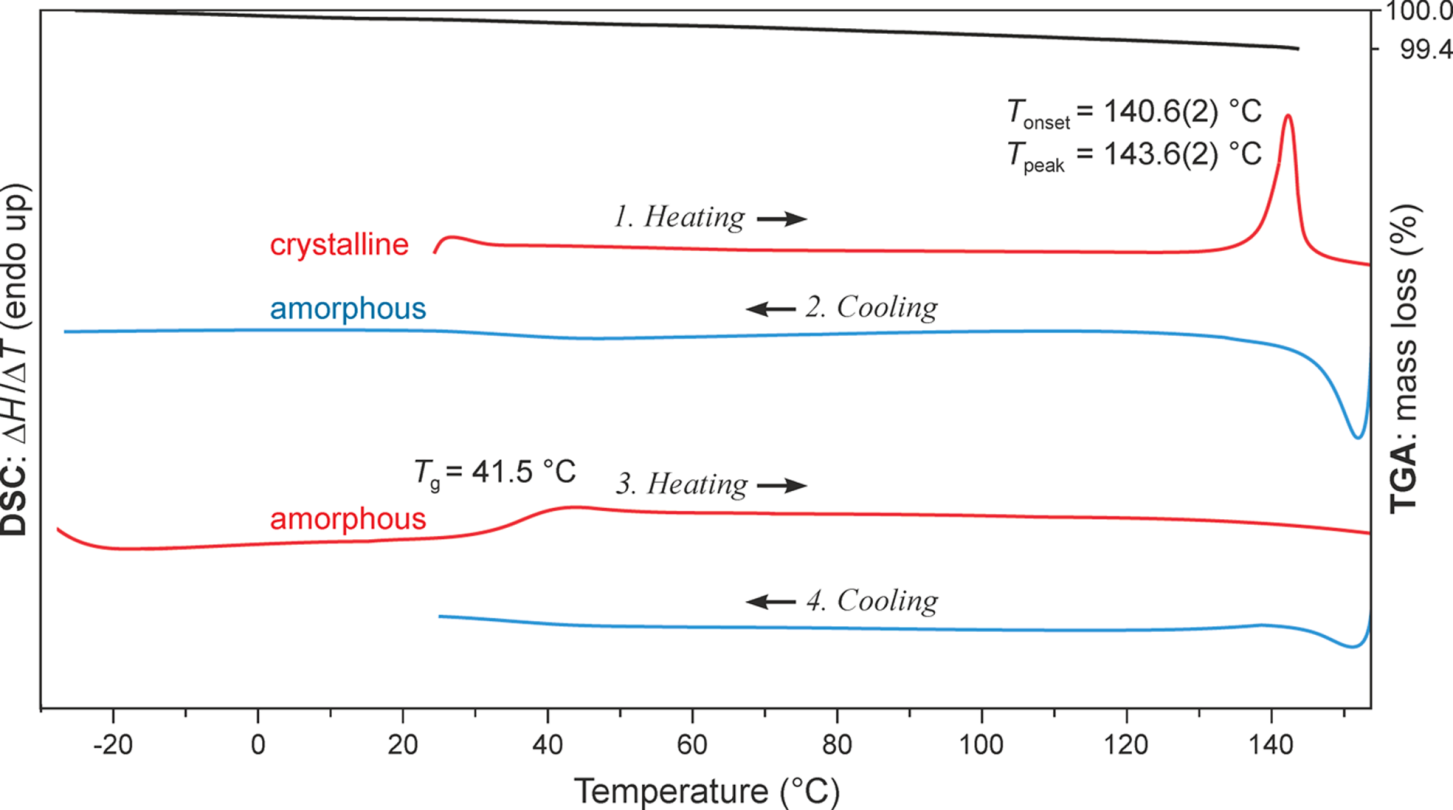
DSC curves: two heating/cooling cycles (red/blue; *T*_g_ = glass transition tem­per­a­ture) and the TGA curve (black) of crystalline sparsentan.

To investigate the thermal properties of the title com­pound, including its glass transition tem­per­a­ture (*T*_g_), the differential scanning calorimetry (DSC) analysis was performed as a heating–cooling–heating cycle. The transition tem­per­a­ture *T*_g_ was determined using a melt–quench technique. The material was first heated beyond its melting point and then cooled rapidly to trap the molten state in an amorphous form. The DSC plot (Fig. 8 ▸, first curve) shows a sharp endothermic peak at 140.6 ± 0.2 °C (onset), which corresponds to the melting of SST, and the TGA thermogram (Fig. 8 ▸) shows a concurrent mass loss of 0.60% between 25 and 145 °C. Following the first heating, the amorphous sample was cooled to −20 °C and then reheated to 155 °C, and during this step, the glass transition was observed at *T*_g_ = 41.5 °C. This relatively low *T*_g_ is indicative of the poor physical stability of amorphous SST and a likelihood of recrystallization from the amorphous state during long-term storage.

#### FT–IR spectroscopy

**Figure 9:**
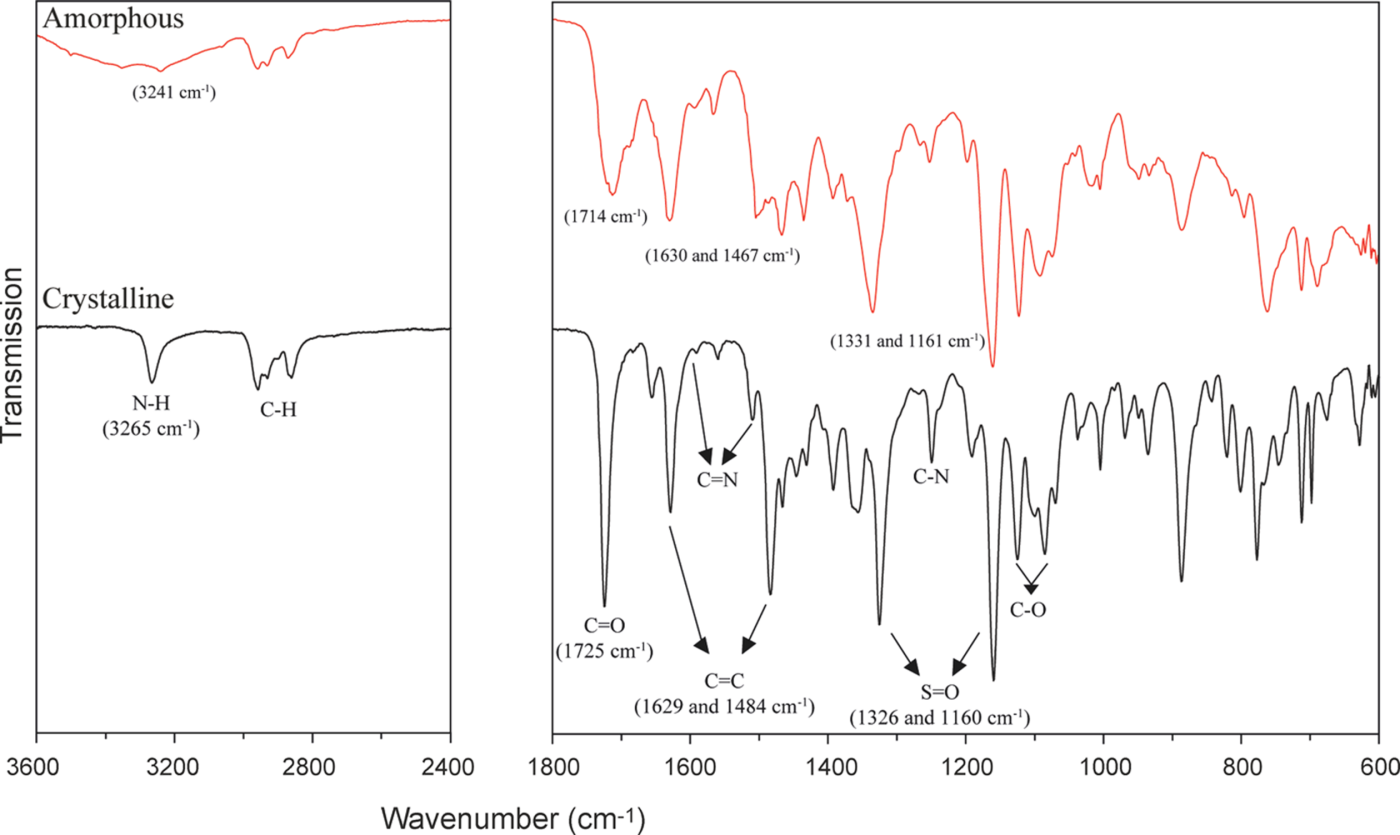
FT–IR spectra of the amorphous (red) and crystalline (black) forms of SST with assigned band positions.

The FT–IR spectra (Fig. 9 ▸) of the amorphous and the crystalline forms of SST were recorded and com­pared. In the case of the crystalline form, sharp peaks were observed at 1725 (C=O), 1629 and 1484 (C=C), and 1326 and 1160 cm^−1^ (O=S=O). In the spectrum of the amorphous form, the corresponding peak positions are shifted by less than 20 cm^−1^ to lower or higher wavenumbers. Additionally, the crystalline form produces a sharp N—H stretching peak above 3000 cm^−1^, whilst the corresponding peak in the spectrum of the amorphous form is significantly broadened. This broadening and shifting of peaks is attributed to mol­ecular rearrangements during the amorphization process which disrupt the periodic order of the crystal lattice in the solid state (Moinuddin *et al.*, 2020 ▸).

## Conclusions

In the solid state, the sparsentan mol­ecule exhibits two dis­or­der­ed chain sections (**A** and **B**), in addition to a dis­or­der­ed cyclo­pentyl ring (section **C**). This study demonstrates the application of com­plementary energy calculations to improve and inter­pret a com­plex disorder model. It was found that an initial disorder model for section **B** contained a minor-occupancy com­ponent with an unviable chain geometry. This led to the establishment of an alternative three-com­ponent disorder model for **B**, resulting in significantly improved structure refinement parameters. Energy calculations confirmed the viability of the three chain geometries implied by the final model for section **B**. In addition, these com­putations indicated that the two disordered chains of sections **A** and **B** of the mol­ecule are uncorrelated with regard to their occupancy.

## Supplementary Material

Crystal structure: contains datablock(s) I, global. DOI: 10.1107/S2053229625007181/vx3016sup1.cif

Structure factors: contains datablock(s) I. DOI: 10.1107/S2053229625007181/vx3016Isup2.hkl

PDF file containing supporting information. DOI: 10.1107/S2053229625007181/vx3016sup3.pdf

Supporting information file. DOI: 10.1107/S2053229625007181/vx3016Isup4.cml

CCDC reference: 2480328

## Figures and Tables

**Table 1:** Experimental details

Crystal data	
Chemical formula	C_32_H_40_N_4_O_5_S
*M*_r_	592.74
Crystal system, space group	Triclinic, *P* 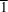
Temperature (K)	193
*a*, *b*, *c* (Å)	11.3363 (10), 11.8815 (8), 14.0763 (10)
α, β, γ (°)	98.113 (6), 112.679 (8), 110.711 (7)
*V* (Å^3^)	1549.4 (2)
*Z*	2
Radiation type	Mo *K*α
μ (mm^−1^)	0.15
Crystal size (mm)	0.25 × 0.25 × 0.15

Data collection	
Diffractometer	Rigaku Xcalibur Gemini ultra diffractometer with a Ruby detector
Absorption correction	Multi-scan (*CrysAlis PRO*; Rigaku OD, 2020 ▸)
*T*_min_, *T*_max_	0.921, 1.000
No. of measured, independent and observed [*I* > 2σ(*I*)] reflections	14469, 6837, 4250
*R*_int_	0.042
(sin θ/λ)_max_ (Å^−1^)	0.641

Refinement	
*R*[*F*^2^ > 2σ(*F*^2^)], *wR*(*F*^2^), *S*	0.054, 0.140, 1.03
No. of reflections	6837
No. of parameters	528
No. of restraints	483
H-atom treatment	H atoms treated by a mixture of independent and constrained refinement
Δρ_max_, Δρ_min_ (e Å^−3^)	0.48, −0.31

Computer programs: *CrysAlis PRO* (Rigaku OD, 2020 ▸), *SHELXT* (Sheldrick, 2015*a* ▸), *SHELXL2014* (Sheldrick, 2015*b* ▸) and *XP* (Bruker, 1998 ▸).

**Table 2:** Hydrogen-bond geometry (Å, °)

*D*—H⋯*A*	*D*—H	H⋯*A*	*D*⋯*A*	*D*—H⋯*A*
N6—H6⋯O11^i^	0.88 (1)	2.12 (1)	2.951 (2)	159 (2)
C28—H28*A*⋯O38^ii^	0.99	2.40	3.318 (3)	153
C28—H28*B*⋯O8^i^	0.99	2.46	3.265 (3)	138

Symmetry codes: (i) 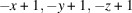; (ii) 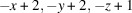.

**Table 3:** Lattice energy differences (PBE-MBD*, kJ mol^−1^) among the six ordered STU structure models and rmsd_15_ values (Å)

Structure	Δ*E*_latt_	rmsd_15_	Structure	Δ*E*_latt_	rmsd_15_
**A1B1**	0.00	0.15	**A2B1**	7.23	0.24
**A1B2**	6.45	0.12	**A2B2**	11.67	0.18
**A1B3**	6.01	0.39	**A2B3**	8.88	0.39
