# Supplementary material for: The disordered structure of sparsentan: energy calculations for com­peting chain con­for­mations
Source: Acta Crystallogr C Struct Chem. 2025 Aug 26;81(Pt 9):530–8. doi: 10.1107/S2053229625007181 (PMC12406262; doi:10.1107/S2053229625007181)
Supplement: Supplementary file 3 [file c-81-00530-sup3.pdf]

Supporting information

The disordered structure of sparsentan: energy calculations for competing chain conformations

Authors

Thomas Gelbrich[ab]*, Kristaps Saršūns[ab] and Doris E. Braun[ab]*

[a] Christian Doppler Laboratory for Advanced Crystal Engineering Strategies in Drug Development, Innrain 52c, Innsbruck, 6020, Austria

[b]Institute of Pharmacy, University of Innsbruck, Innrain 52c, Innsbruck, 6020, Austria

Correspondence email: thomas.gelbrich@uibk.ac.at; doris.braun@uibk.ac.at

Funding information Christian Doppler Forschungsgesellschaft (award to Doris E. Braun).

Table of contents

S1. Database survey 3

S2. Instrumentation used for the characterization of the crystalline phase 7

S3. Pairwise intermolecular energy calculations 9

S4. Disorder 13

S1. Database survey

A search of the Cambridge Structural Database (Groom *et al.*, 2016) for crystal structures containing the *N*-(1,2-oxazol-3-yl)benzenesulfonamide fragment yielded 67 such fragments which are contained in 55 unique crystal structures. For each of these examples, the torsion angles τ_1, τ_2, τ_3, defined in Figure S1, were recorded. Their values, along with the corresponding parameters for the sparsentan (SST) molecule are listed in Table S1. For the diagrams in Figures S2 and S3, τ_2 was plotted against τ_1 and τ_3, respectively. They accompany the τ_1/τ_3 plot shown in Figure 2 and discussed in section 3 of the paper.

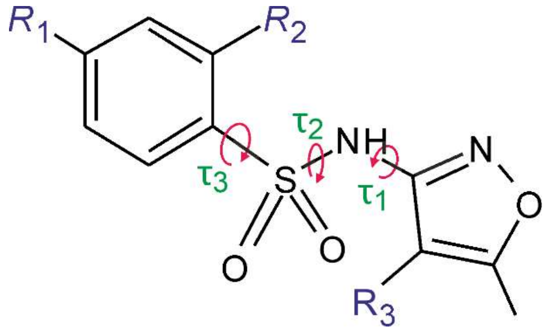

Figure S1 Definition of the torsion angles τ_1, τ_2, τ_3 in the *N*-(1,2-oxazol-3-yl)benzenesulfonamide fragment ($R_2 = R_3$= H, except for SST). (Mackenzie *et al.*, 2017)

Table S1 Torsion angles τ_1, τ_2, τ_3 (°) present in the N-(1,2-oxazol-3-yl)benzenesulfonamide fragment of SST and another 67 examples reported in Cambridge Structural Database.

Compound / CSD refcode	τ_1	τ_2	τ_3 [a]	
SST	-3.1	60.4	41.7	b, c
ALOSEA	-168.3	86.3	82.5	b
AMUTIN	-144.3	59.3	78.4	
BAVZUW	-151.7	65.7	87.1	
CIDDAY	26.4	64.6	86.1	
DITGAQ	-141.3	57.6	81.9	b
DITGAQ01	-141.8	58.5	81.5	b
EQINEZ	-146.9	63.2	70.4	b
EZEZAN	-137.8	50.1	66.1	
FOLDOB	-144.5	73.4	78.2	b
GAQDIN	-144.2	73.9	77.5	
GEBVIW	-148.6	68.3	74.6	b
GEYYOB	-170.4	68.6	48.3	
GOGLAS	25.3	61.5	71.2	b
GOGLEW	-152.4	74.0	80.6	

GOGLEW	-151.5	73.3	80.6	b
GUSHAD01	12.7	58.9	50.8	b
GUSHAD01	-130.8	64.3	45.0	b
JAWKUQ	-145.3	66.5	83.6	b
JAWLAX	-148.0	68.8	64.3	
JAWLAX	-150.5	69.1	64.8	
JAWLAX	-154.7	75.2	64.5	b
JEDZOL	-148.0	54.8	69.3	b
KECXID	25.3	57.6	51.8	
KECXID01	-146.9	60.6	77.5	
KEYGED	13.9	63.4	75.6	b
KEYGED	-26.1	79.4	-74.1	b
LAPCEM	-145.2	61.3	69.4	b
MULMAJ	-146.3	68.6	74.9	
NULZEC	-145.2	58.3	57.6	
OKEWOS	-155.4	76.7	79.7	b
OZOLOF	-160.6	69.7	87.3	
PEJDOA	-150.4	62.0	69.5	
POPJUA	-147.9	64.4	75.3	
POPJUA	-177.6	74.0	80.1	b
POPJUA01	-152.9	72.0	73.6	
QIBCEM	-173.6	69.7	71.5	
QIBCIQ	-153.1	65.7	54.0	
QIBCIQ	-176.5	62.2	51.3	
QIBCIQ	-151.7	67.3	52.2	b
QIBCIQ	-175.9	58.7	50.7	b
QIBCOW	19.6	67.3	-84.7	b
QIBCOW	19.0	66.5	-85.0	
QIBCUC	0.4	71.5	71.0	b
RISZAV	-158.6	73.7	81.9	b
SADHEM	-142.3	65.9	81.4	b
SECGEQ	30.7	57.0	70.5	
SECGIU	-158.1	78.8	-70.6	
SIMJEE	57.2	71.2	-73.2	b
SLFNMB01	-140.8	56.1	76.6	b
SLFNMB02	-150.8	61.6	78.6	b
SLFNMB05	-152.4	61.1	70.3	
SLFNMB06	-161.6	65.7	72.7	b
SUYZIW	-143.7	69.4	76.5	
TAPBUL	-167.5	68.0	69.1	b

TAPCEW	-169.9	58.4	64.1	
TAPGAW	-167.9	74.9	61.6	b
TUJPEV	-164.5	66.2	44.7	b
XAZYAA	-146.5	67.9	69.8	
ZOXVUF	-143.9	70.7	86.1	
ZULGEU01	-152.0	69.6	86.5	b
ZULGEU02	-146.0	62.0	53.2	b
ZULGEU03	-149.1	65.8	78.0	
ZULGEU04	-21.5	81.3	-79.0	
ZULGEU04	23.9	60.5	-80.5	
ZULGEU05	-150.9	62.3	62.7	
ZULGEU05	-177.1	75.5	79.8	
SOFMEJ	-147.9	66.3	77.1	

[a] The twist of the phenyl angle of the ring against the sulfonamide group can be by either of two complementary torsion angles S–C–C–C angles whose absolute values add up to 180°. Of these, the angle with the smallest absolute value was chosen as τ_3.

[b] The structure model as reported in the CSD was inverted so that $\tau_2 \geq 0°$.

[c] This work.

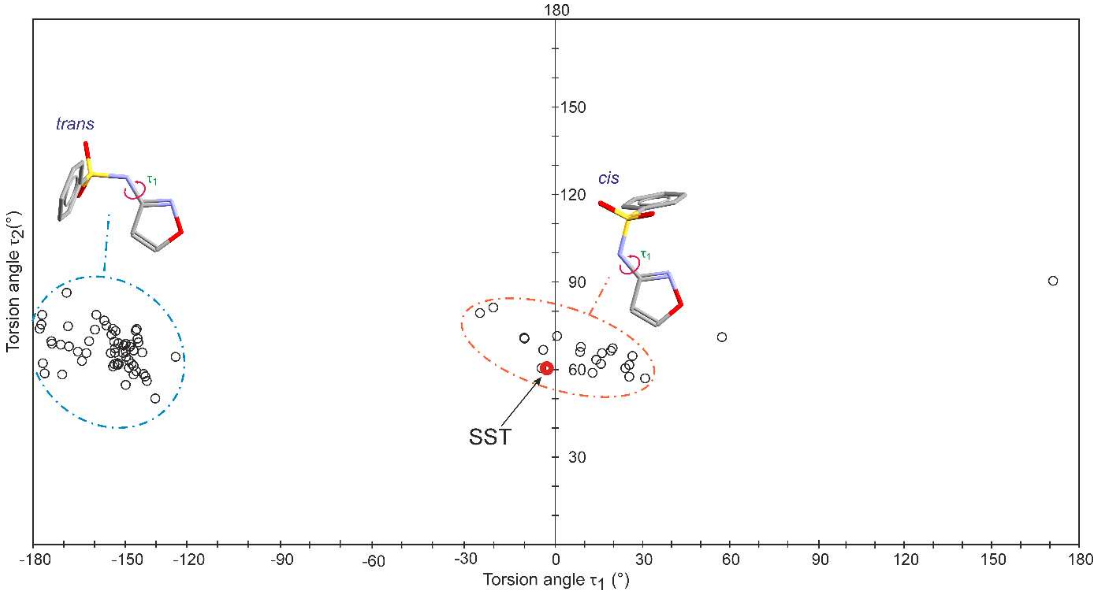

Figure S2 Diagram showing τ_2 *vs.* τ_1 for a survey of experimental conformations of the *N*-(1,2-oxazol-3-yl)benzenesulfonamide fragment. The plot shows two clusters, each corresponding to a conformation.

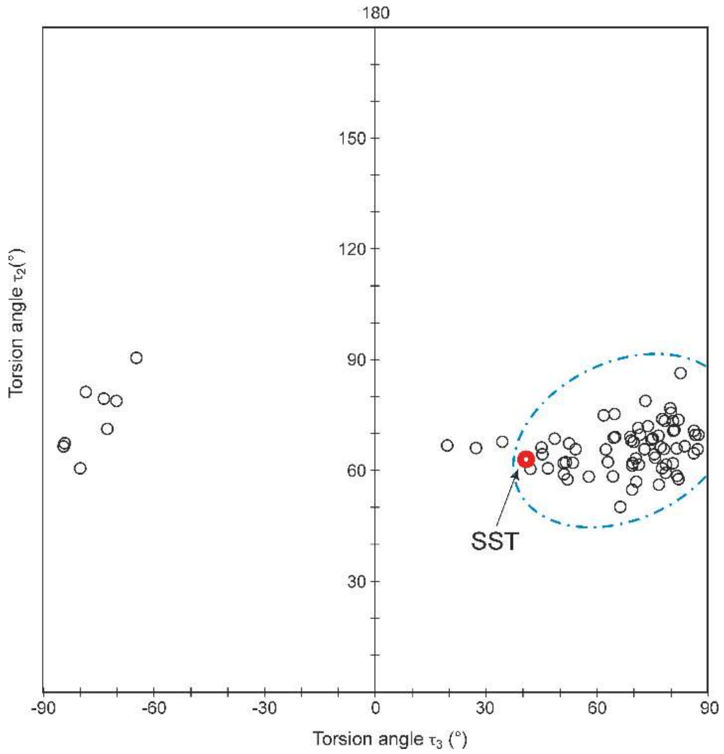

Figure S3 Diagram showing τ_2 *vs.* τ_3 for a survey of experimental conformations of the *N*-(1,2-oxazol-3-yl)benzenesulfonamide fragment. Most data points, including the data point for SST are located in dense cloud where 50° < τ_2 < 85° and 40° < τ_3 < 90°.

S2. Instrumentation used for the characterization of the crystalline phase

The powder X-ray diffraction (PXRD) patterns were measured at ambient temperature on an *X'Pert PRO diffractometer* (PANalytical, Almelo, NL) using copper radiation source (Cu$K\alpha_{1,2}$) at the wavelength 1.54190 Å, equipped with a θ/θ coupled goniometer in transmission geometry and programmable XYZ stage with well plate holder. The setup featured a focusing 0.5° divergence slit and a 0.02° Soller slit collimator on the incident beam side. On the diffracted beam side, there was a 2 mm antiscattering slit and a 0.02° Soller slit collimator, along with a solid-state *PIXcel^{1D}* detector. The tube voltage and current were set to 40 kV and 40 mA. The diffraction patterns were recorded using a stepsize of 2θ = 0.013° with 40 s per step in the 2θ range between 2° to 40°.

Thermogravimetric analysis was carried out with a TGA7 thermogravimeter (PerkinElmer, Norwalk, Connecticut), controlled by the Pyris 8.0 software, with approximately 3 mg of substance. A two-point calibration of the temperature was performed with ferromagnetic materials (Alumel and Ni, Curie-point standards, PerkinElmer). A heating rate of 10 °C min^{-1} was applied, and N_2 was used as purge gas (sample purge: 20 mL min^{-1}, balance purge: 40 mL min^{-1}).

Differential scanning calorimetry (DSC) was performed with a DSC 204 F1 - Phoenix (Netzsch, Selb, Germany) using the Netzsch Proteus 7 software. Approximately 3 mg sample were weighed into Al pans (25 µL) and sealed with a cover, the analysis employed a heating rate of 10 °C min^{-1}, with dry nitrogen purge gas (sample purge: 20 mL min^{-1}, protective purge: 40 mL min^{-1}). The temperature calibration of the instrument was carried out using pure cyclohexane (melting point = –87.0 °C), mercury (–38.8°C), benzophenone (48.0 °C), indium (156.6 °C) and caffeine (236.2 °C), while sensitivity calibration was performed with cyclohexane (heat of fusion = 79.4 J g^{-1}), mercury (11.4 J g^{-1}), indium (28.45 J g^{-1}), tin (60.5 J g^{-1}) and bismuth (–53.1 J g^{-1}). The stated (extrapolated onset) temperatures have an error calculated at 95% Cl and are based on at least three measurements.

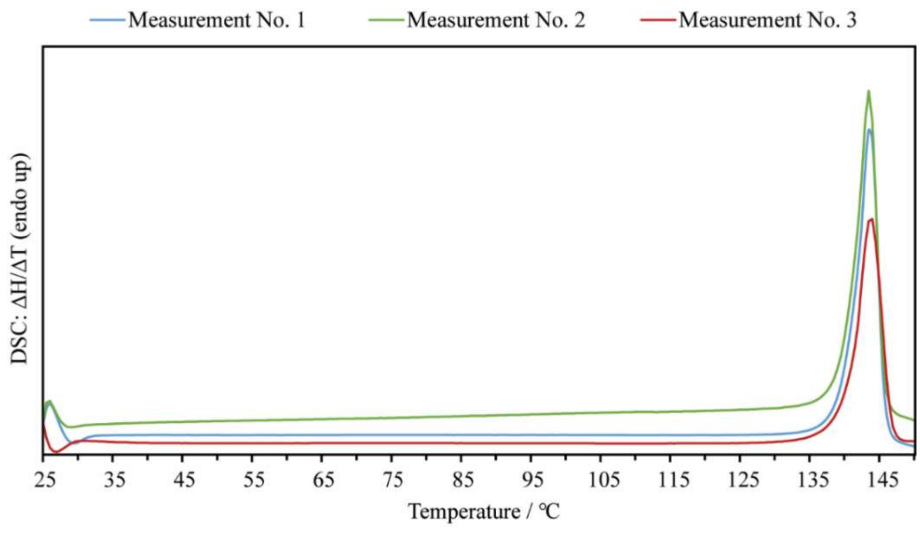

Figure S4 DSC curves of the crystalline sparsentan.

Table S2 Obtained temperature data (°C) from DSC measurements – (calculation of error).

Measurement no.	Temperature	Average	Standard deviation	Result
Calculation of error (T_{onset})				
1	140.7			
2	140.6	140.6	0.2	140.6 ± 0.2
3	140.4			
Calculation of error (T_{peak})				
1	143.8			
2	143.6	143.6	0.2	143.6 ± 0.2
3	143.5			

Infrared spectra were recorded with a diamond ATR (PIKE GaldiATR, Madison, US) crystal on a Bruker Vertex 70 FTIR spectrometer (Bruker Analytische Messtechnik GmbH, Germany). The spectra were recorded between 4000 and 400 cm^{-1} at an instrument resolution of 2 cm^{-1}, with 32 scans per spectrum.

S3. Pairwise intermolecular energy calculations

Table S3 Pairwise intermolecular interactionsa seen in SST model **A1B1**.

No.	Symop.	*n*	R / Å	E_E	E_P	E_R	E_D	E_{tot}	E_{inter}
					/ kJ mol^{-1}				
1	*-x, -y, -z*	1	7.74	−99.5	−31.1	−91.4	114.3	−137.2	−68.6
2	*-x, -y, -z*	1	9.05	−40.8	−12.4	−91.3	86.3	−78.5	−39.25
3	*-x, -y, -z*	1	8.42	−28.8	−5.6	−94.1	69.2	−73.8	−36.9
4	*-x, -y, -z*	1	9.65	−34.4	−9.0	−40.0	46.2	−49.3	−24.65
5	*-x, -y, -z*	1	9.66	−22.3	−4.8	−39.4	33.4	−40.7	−20.35
6	*x, y, z*	2	11.32	−9.6	−4.8	−39.9	18.6	−36.9	−36.9
7	*-x, -y, -z*	1	13.96	−11.1	−5.2	−17.3	11.5	−23.6	−11.8
8	*-x, -y, -z*	1	11.84	−4.2	−0.2	−25.5	14.9	−17.6	−8.8
9	*x, y, z*	2	13.04	3.4	−2.6	−28.6	16.8	−12.9	−12.9
10	*x, y, z*	2	11.88	−1.7	−0.7	−16.9	6.7	−12.8	−12.8
11	*-x, -y, -z*	1	16.91	−1.7	−0.1	−12.8	7.8	−8.2	−4.1

[a] electrostatic (E_E), polarization (E_P), dispersion (E_D), and exchange-repulsion (E_R). $E_{tot} = k_E E_E + k_P E_P + k_D E_D + k_R E_R$, with k being scale factors (Mackenzie *et al.*, 2017). E_{inter} was calculated as follows: $E_{tot}/2 * n$.

Table S4 Pairwise intermolecular interactionsa seen in SST model **A1B2**.

No.	Symop.	*n*	R / Å	E_E	E_P	E_R	E_D	E_{tot}	E_{inter}
					/ kJ mol^{-1}				
1	*-x, -y, -z*	1	7.73	−97.0	−29.8	−89.6	111.2	−134.0	−67.0
2	*-x, -y, -z*	1	8.98	−43.8	−13.3	−87.6	82.6	−81.4	−40.7
3	*-x, -y, -z*	1	8.33	−32.6	−8.3	−101.1	77.3	−80.8	−40.4
4	*-x, -y, -z*	1	9.81	−32.9	−9.1	−37.6	43.3	−47.5	−23.75
5	*x, y, z*	2	11.33	−8.5	−4.3	−39.3	18.9	−34.7	−34.7
6	*-x, -y, -z*	1	9.77	−17.6	−3.3	−38.7	33.6	−34.0	−17.0
7	*-x, -y, -z*	1	13.94	−13.3	−5.2	−18.1	13	−25.7	−12.85
8	*-x, -y, -z*	1	11.86	−5.7	−0.3	−22.2	19.3	−13.7	−6.85
9	*x, y, z*	2	11.83	−2.1	−0.6	−16.4	5.5	−13.5	−13.5
10	*x, y, z*	2	13.15	3.7	−2.4	−27.7	15.8	−12.1	−12.1
11	*-x, -y, -z*	1	16.94	−1.7	−0.1	−12.5	7.6	−8.1	−4.05

[a] electrostatic (E_E), polarization (E_P), dispersion (E_D), and exchange-repulsion (E_R). $E_{tot} = k_E E_E + k_P E_P + k_D E_D + k_R E_R$, with k being scale factors (Mackenzie *et al.*, 2017). E_{inter} was calculated as follows: $E_{tot}/2 * n$.

Table S5 Pairwise intermolecular interactionsa seen in SST model **A1B3**.

**No.**	**Symop.**	***n***	**R / Å**	$\boldsymbol{E_E}$	$\boldsymbol{E_P}$	$\boldsymbol{E_R}$ / kJ mol^{-1}	$\boldsymbol{E_D}$	$\boldsymbol{E_{tot}}$	$\boldsymbol{E_{inter}}$
1	*-x, -y, -z*	1	6.65	−99.2	−31.3	−121.6	135.9	−150.1	−75.05
2	*-x, -y, -z*	1	7.94	−35.0	−9.3	−88.2	62.1	−82.3	−41.15
3	*-x, -y, -z*	1	9.88	−41.8	−13.7	−86.1	87.9	−74.9	−37.45
4	*-x, -y, -z*	1	9.85	−38.7	−9.0	−57.1	56.5	−62.4	−31.2
5	*x, y, z*	2	11.46	−9.0	−4.7	−35.3	18.5	−32.3	−32.3
6	*-x, -y, -z*	1	13.63	−14.3	−5.1	−18.0	13.1	−26.6	−13.3
7	*-x, -y, -z*	1	10.25	−9.6	−2.9	−25.1	18.2	−22.9	−11.45
8	*x, y, z*	2	13.79	−2.5	−0.2	−16.0	7.8	−12.0	−12.0
9	*x, y, z*	2	13.52	5.2	−2.6	−23.1	11.4	−9.5	−9.5
10	*x, y, z*	2	11.77	−2.6	−0.4	−10.1	4.3	−9.2	−9.2
11	*-x, -y, -z*	1	18.17	−1.4	−0.1	−10.0	6.3	−6.4	−3.2
12	*x, y, z*	2	13.9	−2.4	−0.6	−6.7	6.3	−4.9	−4.9
13	*x, y, z*	2	16.82	0.5	−0.1	−2.9	0.5	−1.8	−1.8

[a] electrostatic (E_E), polarization (E_P), dispersion (E_D), and exchange-repulsion (E_R). $E_{tot} = k_E E_E + k_P E_P + k_D E_D + k_R E_R$, with *k* being scale factors (Mackenzie *et al.*, 2017). E_{inter} was calculated as follows: $E_{tot}/2 * n$.

Table S6 Pairwise intermolecular interactionsa seen in SST **A2B1**.

**No.**	**Symop.**	***n***	**R / Å**	$\boldsymbol{E_E}$	$\boldsymbol{E_P}$	$\boldsymbol{E_R}$ / kJ mol^{-1}	$\boldsymbol{E_D}$	$\boldsymbol{E_{tot}}$	$\boldsymbol{E_{inter}}$
1	-x, -y, -z	1	7.64	−101.2	−31.5	−85.1	117.1	−132.2	−66.1
2	-x, -y, -z	1	9.22	−36.8	−9.6	−109.9	90.1	−86.1	−43.05
3	-x, -y, -z	1	8.64	−28.9	−5.3	−93.6	69.8	−72.9	−36.45
4	-x, -y, -z	1	9.83	−30.3	−8.0	−38.4	38.1	−47.8	−23.9
5	-x, -y, -z	1	9.64	−20.5	−4.8	−40.7	35.1	−39.1	−19.55
6	x, y, z	2	11.21	−8.8	−4.0	−36.5	17.0	−33.6	−33.6
7	-x, -y, -z	1	13.89	−9.6	−5.3	−16.7	8.7	−23.2	−11.6
8	-x, -y, -z	1	11.62	−3.9	−0.3	−25.5	13.2	−18.4	−9.2
9	x, y, z	2	11.88	−1.7	−0.5	−16.2	6.2	v12.5	−12.5
10	x, y, z	2	13.12	3.5	−2.3	−26.9	14.9	−12.3	−12.3
11	-x, -y, -z	1	16.85	−2.0	−0.1	−15.2	8.9	−9.9	−4.95
12	-x, -y, -z	1	17.82	0.7	−0.1	−1.0	0.0	−0.2	−0.1

[a] electrostatic (E_E), polarization (E_P), dispersion (E_D), and exchange-repulsion (E_R). $E_{tot} = k_E E_E + k_P E_P + k_D E_D + k_R E_R$, with *k* being scale factors (Mackenzie *et al.*, 2017). E_{inter} was calculated as follows: $E_{tot}/2 * n$.

Table S7 Pairwise intermolecular interactionsa seen in SST **A2B2**.

No.	Symop.	*n*	R / Å	E_E	E_P	E_R	E_D	E_{tot}	E_{inter}
						/ kJ mol^{-1}			
1	-x, -y, -z	1	7.66	−100.7	−30.5	−82.0	117.7	−127.8	−63.9
2	-x, -y, -z	1	9.05	−39.9	−12.7	−110.3	89.2	−92.5	−46.25
3	-x, -y, -z	1	8.46	−32.4	−7.9	−103.3	79.7	−80.8	−40.4
4	-x, -y, -z	1	10.02	−28.9	−8.0	−35.8	35.8	−45.5	−22.75
5	-x, -y, -z	1	9.76	−19.6	−3.7	−38.7	36.9	−34.3	−17.15
6	x, y, z	2	11.25	−7.5	−3.2	−34.9	16.9	−30.2	−30.2
7	-x, -y, -z	1	13.88	−12.4	−5.3	−18.0	11.2	−25.9	−12.95
8	x, y, z	1	11.77	−2.2	−0.5	−16.5	5.7	−13.5	−13.5
9	-x, -y, -z	2	11.67	−5.5	−0.4	−21.5	18.3	−13.5	−6.75
10	x, y, z	2	13.22	3.6	−2.2	−26.9	14.6	−12.3	−12.3
11	-x, -y, -z	1	16.90	−2.6	−0.1	−14.9	10.7	−9.2	−4.6
12	-x, -y, -z	1	17.45	0.7	−0.1	−1.2	0.1	−0.3	−0.15

[a] electrostatic (E_E), polarization (E_P), dispersion (E_D), and exchange-repulsion (E_R). $E_{tot} = k_E E_E + k_P E_P + k_D E_D + k_R E_R$, with k being scale factors (Mackenzie *et al.*, 2017). E_{inter} was calculated as follows: $E_{tot}/2 * n$.

Table S8 Pairwise intermolecular interactionsa seen in SST **A2B3**.

No.	Symop.	*n*	R / Å	E_E	E_P	E_R	E_D	E_{tot}	E_{inter}
						/ kJ mol^{-1}			
1	-x, -y, -z	1	6.60	−104.4	−32.3	−109.4	142	−141.8	−70.9
2	-x, -y, -z	1	9.83	−47.6	−13.1	−113	116.6	−86.4	−43.2
3	-x, -y, -z	1	8.06	−33.7	−9.2	−85.1	60.0	−79.6	−39.8
4	-x, -y, -z	1	10.07	−34.8	−7.5	−58.0	51.6	−60.9	−30.45
5	x, y, z	2	11.37	−7.2	−3.5	−32.2	15.8	−28.5	−28.5
6	-x, -y, -z	1	13.56	−14.1	−5.4	−18.5	12.3	−27.4	−13.7
7	-x, -y, -z	1	10.19	−6.7	−2.4	−27.4	15.8	−22.9	−11.45
8	x, y, z	2	13.76	−3.9	−0.3	−17.4	11.8	−12.2	−12.2
9	x, y, z	2	13.58	5.4	−2.4	−24.1	11.9	−9.7	−9.7
10	x, y, z	2	11.69	−2.6	−0.3	−9.7	4.5	−8.6	−8.6
11	-x, -y, -z	1	18.08	−1.9	−0.1	−12.7	8.4	−8.0	−4.0
12	x, y, z	2	14.19	−1.7	−0.5	−5.9	4.2	−4.7	−4.7
13	x, y, z	2	16.94	−0.1	−0.1	−6.2	3.4	−3.5	−3.5
14	-x, -y, -z	1	18.74	0.1	0	−1.0	0	−0.8	−0.4
15	-x, -y, -z	1	17.59	0.6	−0.1	−1.1	0	−0.3	−0.15

[a] electrostatic (E_E), polarization (E_P), dispersion (E_D), and exchange-repulsion (E_R). $E_{tot} = k_E E_E + k_P E_P + k_D E_D + k_R E_R$, with k being scale factors (Mackenzie *et al.*, 2017). E_{inter} was calculated as follows: $E_{tot}/2 * n$.

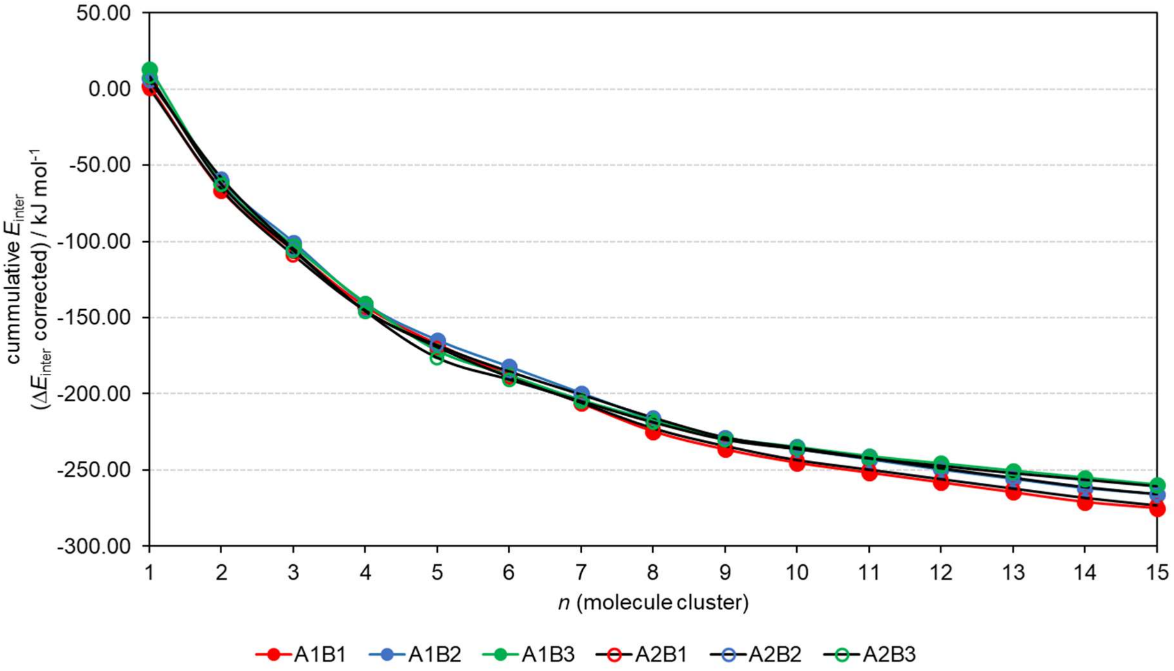

Figure S5 Cumulative sum of pairwise intermolecular interactions in sparsentan structure models A1B1 to A2B3. Note that the curves are offset by the ΔE_{intra} values of the conformations present in the structure models.

S4. Disorder

Table S9 Torsion angles (°) for disorder section A.

Torsion angle	Component A1	Component A2
N29–C33–C39–C40	−81.1(5)	−143.0(2)
C33–C39–C40–C41	−178.9(4)	−166(2)
C39–C40–C41–C42	−67.0(5)	−155(2)
Conformation	*gauche*/*trans*/*gauche*	all-*trans*
Occupancy	0.804(5)	0.196(5)

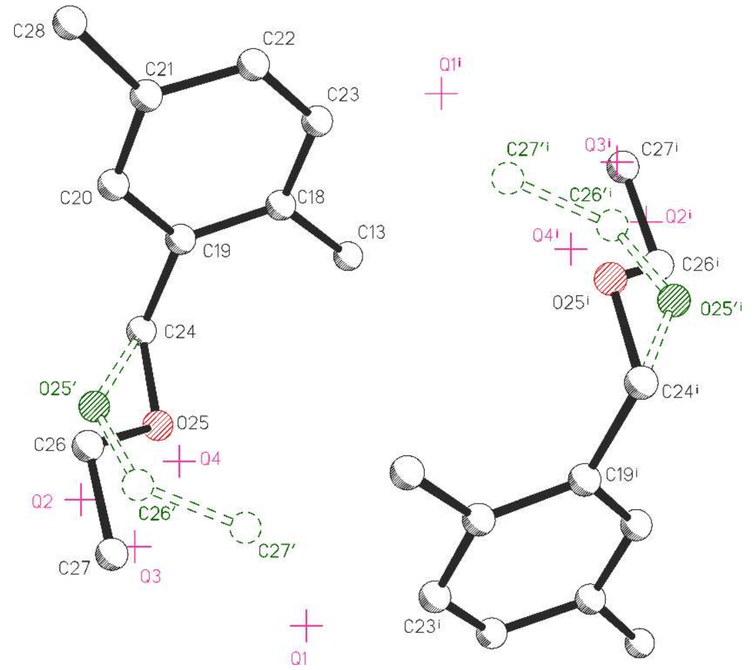

Figure S6 Initial two-component disorder model for section B of the SST molecule, featuring an unusually large separation between the alternative positions of methyl group C27···C27' = 1.94 Å, a C27' methyl group in unreasonably close proximity to the phenyl ring of a second molecule (C27'···C23^{i} = 2.63 Å). In addition, four significant rest peaks are located in the region of the disordered chain, in particular Q1 (1.02 e Å^{-3}) at a distance of 2.64 Å from C23^{i}. Symmetry code (i) $1 - x$, $2 - y$, $1 - z$.

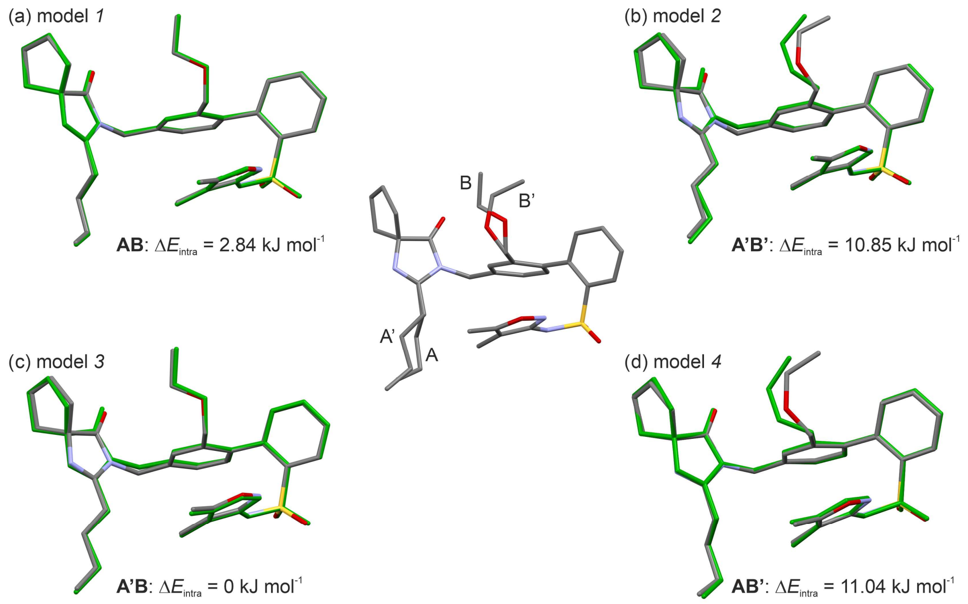

Figure S7 The initial two-component disorder model for sections A/A' and B/B': overlay of the experimental conformations (coloured by element) with the conformations observed in the PBE-TS structures (Tkatchenko & Scheffler, 2009). Intramolecular energy differences ($\Delta E_{\mathrm{intra}}$) were calculated relative to the lowest energy among the four conformations. The comparison of (a) with (b) and (c) with (d) shows that the initial conformation B' results in a significantly poorer fit and an increased $\Delta E_{\mathrm{intra}}$ value.

Table S10 Torsion angles (°) in the final three-way split disorder model for section B.

Torsion angle	Component B1	Component B2	Component B3
C18–C19–C24–O25	−86.1(7)	−130.2(17)	−71.6(10)
C19–C24–O25–C26	−75.1(10)	−74(2)	174.6(11)
C24–O25–C26–C27	−179.8(9)	−176(3)	−86(3)
Occupancy	0.597(3)	0.223(3)	0.180(2)

[a] Torsion angles C18–C23–C24B–O25B, C23–C24B–O25B–C26B, C24B–O25B–C26B–C27B

References

Groom, C. R., Bruno, I. J., Lightfoot, M. P. & Ward, S. C. (2016). *Acta Cryst. B* **72**, 171-179.
Mackenzie, C. F., Spackman, P. R., Jayatilaka, D. & Spackman, M. A. (2017). *IUCrJ* **4**, 575-587.
Tkatchenko, A. & Scheffler, M. (2009). *Phys. Rev. Lett.* **102**, 073005.
